# Association analysis of rice resistance genes and blast fungal avirulence genes for effective breeding resistance cultivars

**DOI:** 10.3389/fmicb.2022.1007492

**Published:** 2022-11-09

**Authors:** Dagang Tian, Yun Deng, Xiaoshuang Yang, Gang Li, Qixiang Li, Haiying Zhou, Ziqiang Chen, Xinrui Guo, Yan Su, Yuming Luo, Liming Yang

**Affiliations:** ^1^Biotechnology Research Institute, Fujian Academy of Agricultural Sciences, Fuzhou, China; ^2^Nanping Institute of Agricultural Sciences, Sanming Agricultural Bureau, Fujian, China; ^3^Jiangsu Collaborative Innovation Center of Regional Modern Agriculture and Environmental Protection, Jiangsu Key Laboratory for Eco-Agricultural Biotechnology Around Hongze Lake, Huaiyin Normal University, Huai’an, China; ^4^College of Biology and the Environment, Nanjing Forestry University, Nanjing, China

**Keywords:** rice blast, *Magnaporthe oryzae*, avirulence gene, resistance gene, resistance breeding

## Abstract

Utilization of rice blast-resistance (*R*) genes is the most economical and environmentally friendly method to control blast disease. However, rice varieties with *R* genes influence the outcome of genetic architectures of *Magnaporthe oryzae* (*M. oryzae*), and mutations in avirulence (*AVR*) genes of *M. oryzae* may cause dysfunction of the corresponding *R* genes in rice varieties. Although monitoring and characterizing rice *R* genes and pathogen *AVR* genes in field populations may facilitate the implementation of effective *R* genes, little is known about the changes of *R* genes over time and their ultimate impact on pathogen *AVR* genes. In this study, 117 main cultivated rice varieties over the past five decades and 35 *M. oryzae* isolates collected from those diseased plants were analyzed by PCR using gene-specific markers of the nine *R* genes and six primer pairs targeting the coding sequence or promoter of *AVR* genes, respectively. The *R* genes *Pigm, Pi9*, *Pi2*, *Piz-t*, *Pi-ta*, *Pik, Pi1*, *Pikp,* and *Pikm* were identified in 5, 0, 1, 4, 18, 0, 2, 1, and 0 cultivars, respectively. Significantly, none of these *R* genes had significant changes that correlated to their application periods of time. Among the four identified *AVR* genes, *AVR-Pik* had the highest amplification frequency (97.14%) followed by *AVR-Pita* (51.43%) and *AVR-Pi9* (48.57%); *AVR-Piz-t* had the lowest frequency (28.57%). All these *AVR* genes except *AVR-Pi9* had 1–2 variants. Inoculation mono-genic lines contained functional genes of *Pi2/9* and *Pik* loci with 14 representative isolates from those 35 ones revealed that the presence of certain *AVR-Piz-t*, *AVR-Pita* variants, and *AVR-Pik-E + AVR-Pik-D* in *M. oryzae* populations, and these variants negated the ability of the corresponding *R* genes to confer resistance. Importantly, *Pi2, Pi9,* and *Pigm* conferred broad-spectrum resistance to these local isolates. These findings reveal that the complex genetic basis of *M. oryzae* and some effective blast *R* genes should be considered in future rice blast-resistance breeding programs.

## Introduction

Rice is the main staple food crop for more than 50% of the world population. Rice blast disease, caused by *Magnaporthe oryzae* B.C. Couch, is a major threat to rice production in most rice-planting regions. The most efficient tool for rice blast disease control is the implementation of host *R* genes (Velent and Chumley, 1991). In principle, each rice *R* gene has a cognate *AVR* gene in *M. oryzae* (Flor, 1971), the utilization of *R* genes in agriculture has a selective effect on the corresponding *AVR* genes, leading to the rapid evolution of fitness of *AVR* genes toward virulence to their respective *R* genes. Thus, the identification and utilization of *R* genes that confer broad-spectrum resistance against a large number of pathogen races is the most effective approach to manage the disease.

To date, more than 100 blast *R* genes have been identified, and at least 28 have been cloned and characterized (Deng et al., 2017; Meng et al., 2021). *Pigm, Pi9*, *Pi2*, *Piz-t* in the *Pi2/9* locus, and *Pik, Pi1*, *Pikp,* and *Pikm* in the *Pik* locus, which are well-known broad-spectrum resistance *NBS-LRR* (nucleotide-binding site-leucine-rich repeat) genes with good resistance against *M. oryzae* in rice-planting region of southern China (Tian et al., 2020, 2021). With respect to the cognate *AVR* genes, a total of 12 have been cloned: *PWL1* (Kang et al., 1995), *AVR-Pita* (Orbach et al., 2000), *AVR-Pib* (Zhang et al., 2015), *AVR1-Co39* (Farman et al., 2002), *PWL2*, *ACE1* (Böhnert et al., 2004), *AVR-Piz-t* (Li et al., 2019), *AVR-Pii, AVR-Pia*, *AVR-Pik/km/kp* (Yoshida et al., 2009), *AVR-Pi54* (Ray et al., 2016), and *AVR-Pi9* (Wu et al., 2015). In general, there are three modes by which *R* genes recognize their respective *AVR* partners. In the first mode, a single *R* gene recognizes a single *AVR* gene, e.g., *Pi-ta* recognizes *AVR-Pita* (Jia et al., 2000). In the second mode, a single *R* gene recognizes two *AVR* genes, such as *Pia/AVR1-Co39* and *Pia/AVR-Pia* (Farman et al., 2002; Yoshida et al., 2009; Okuyama et al., 2011; Cesari et al., 2013). In the third mode, multiple *R* alleles recognize multiple *AVR* alleles, e.g., the six haplotypes (A–F) of the *AVR-Pik* allele in different isolates can be recognized by *Pik*/*kp*/*km*/*ks*/*kh*, *Pik*/*kp*/*km*/*kh*, and *Pik*/*km*/*kh*, etc (Yoshida et al., 2009; Kanzaki et al., 2012; Wu et al., 2014; Hu et al., 2022). In fact, a rice variety with a particular *R* gene may lose its resistance if a mutation(s) occurs in the corresponding *AVR* gene (Lee and Cho, 1990). Particularly, three *AVR* genes, namely *AVR-Pita*, *AVR-Pik,* and *AVR-Piz-t*, have a relatively large number of polymorphisms, including point mutations, transposon insertions, and partial or total gene deletions, and hence usually escape recognition by the corresponding *R* genes in rice (Dean et al., 2005; Zhou et al., 2007; Chuma et al., 2011; Huang et al., 2014; Zhang et al., 2015). For example, Wang et al. (2020) identified 11 haplotypes encoding three new *AVR-Piz-t* variants in 100 isolates among 312 isolates of *M. oryzae* in Yunnan, China, including a 39-bp deletion and a 192-bp solo-LTR (long terminal repeat) homolog of the retrotransposon inago2 that had inserted into the promoter region and created a frameshift in the coding sequence. Each of these genetic alterations pushed *AVR-Piz-t* to a virulent form. Additionally, Meng et al. (2020) identified 5 virulent isolates of *AVR-Pita* from 202 isolates of *M. oryzae* in Heilongjiang, China. They also found 7 *AVR-Pik* alleles and 4 *AVR-Piz-t* variants among another 335 isolates of *M. oryzae* in Heilongjiang, China, leading to the loss of function of the corresponding *R* genes (Meng et al., 2019). In contrast, variants of *AVR-Pia* and *AVR-Co39*, which recognize the same *R* gene *Pia*, mostly are deletion mutants and thus can be easily lost among isolates of *M. oryzae* (Yoshida et al., 2009; Zheng et al., 2011).

The complex rice-growing environment in southern of China has resulted in a rich genetic diversity and complexity of rice blast fungus, and thus, the disease is prone to recurrence. Therefore, the local varieties as well as those that have come from breeding programs must contain cognate *R* genes to ensure their survival (Tian et al., 2020). To date, no study has correlated the evolution of *AVR* genes in *M. oryzae* with the application of *R* genes in rice. Therefore, to identify effective *R* genes in the specific rice-growing region, we utilized 117 representative cultivars from the main rice-planting regions in South China over the past 50 years to carry out a field evaluation of resistance to rice blast disease and characterize the genetic basis of *R* genes in these rice cultivars and *AVR* genes. The results provide a reference for rational distribution of rice blast *R* genes.

## Materials and methods

### Rice cultivars and *Magnaporthe oryzae* isolates

The cultivars comprised 117 *Oryza sativa* accessions and positive controls including IRBLzt-t (*Piz-t*), C101A51 (*Pi2*), IRBL9-W/75–1-127 (*Pi9*), Gumei 4 (*Pigm*), IRBLta-K1 (*Pi-ta*), IRBLZK-Ka (*Pik*), IRBLZKp-K60 (*Pikp*), IRBL1-CL (*Pi1*), and IRBLKM-Ts (*Pikm*), IRBLkh-K3 (*Pikh*), and IRBLks-F5 (*Piks*) collected from the Fujian Provincial Key Laboratory of Genetic Engineering for Agriculture, Fujian Academy of Agricultural Sciences, Fuzhou, China. The 117 cultivars comprised 27 three-line male sterility lines, 11 two-lined male sterility lines, and 79 restorer lines or conventional varieties. Forty-six accessions were bred in the 2000s, 19 before the 1980s, 16 in the 1980s, 28 in the 1990s, and 8 in the 2010s. These selected cultivars were from large acreages planted in the past several decades. Supplementary Table 1 lists detailed information regarding the accessions.

A total of 35 *M. oryzae* isolates were purified from leaf-diseased of 13 diseased rice cultivars at the mature stage in Shanghang, China, in 2017. Isolate purification was achieved with a single-spore isolation method (Jia et al., 2000). Subsequently, the purified mycelia derived from a single spore were grown on the surface of oatmeal agar medium. The mycelia and spores were allowed to colonize the entire filter paper. Each filter paper with the fungus was then transferred to a distilled envelope and stored at −20°C.

### Field and greenhouse evaluation of blast resistance

The 117 cultivars were planted in the disease nursery beds with 20 plants per plot; the highly susceptible variety Guangluai 4 was sown on the plot borders. Disease severity was scored using leaves of seedlings and at the maximum tillering stage using a 1–9 scale: 0, no symptoms, i.e., a highly resistant (HR) phenotype; 1 and 2, only very small spots on leaves, indicating a resistant (R) phenotype; 3, small elliptical lesions, indicating a moderately resistant (MR) phenotype; 4, an elliptical lesion area of <2% of the entire leaf, indicating a moderately susceptible (MS) phenotype; 5, lesion area < 10%, indicating a susceptible (S) phenotype; 6 and 7, lesion area 10–25% or 26–50%, respectively, indicating susceptible phenotype; 8 and 9, lesion area 51–75% or > 75%, respectively, indicating highly susceptible (HS) phenotype. Scale values of 0, 1, 3, 5, 7, and 9 were used to score disease phenotype on spikes at maturity: 0, no evidence of infection; 1, diseased spike rate of <5%; 3, rate 5%–10%; 5, rate 10%–25%; 7, rate 25%–50%; and 9, rate 50%–100%. The comprehensive resistance evaluation was scored as 25%, 25%, and 50% of the diseased scoring at the seedling stage, maximum tillering stage, and maturity, respectively.

The greenhouse evaluation of blast resistance is shown by Tian et al. (2016). Briefly, the *M. oryzae* isolates were prepared in the dark for approximately 5 to 7 days at 28°C, and then under light for 5 to 7 days at room temperature for sporulation. Twenty rice seedlings of each variety were grown in a greenhouse for about 2 weeks under natural conditions and then were spray inoculated with spores at a concentration of 5 × 10^5^ spores mL^−1^ until run-off. After inoculation, seedlings were maintained in the dark for 24 h and then kept under high humidity for 1 week to evaluate their symptoms. The disease score standard scale is above described. The recurrent parent variety Co39 was susceptibility control.

### DNA extraction, marker evaluation, and genotyping

**Figure 1:**
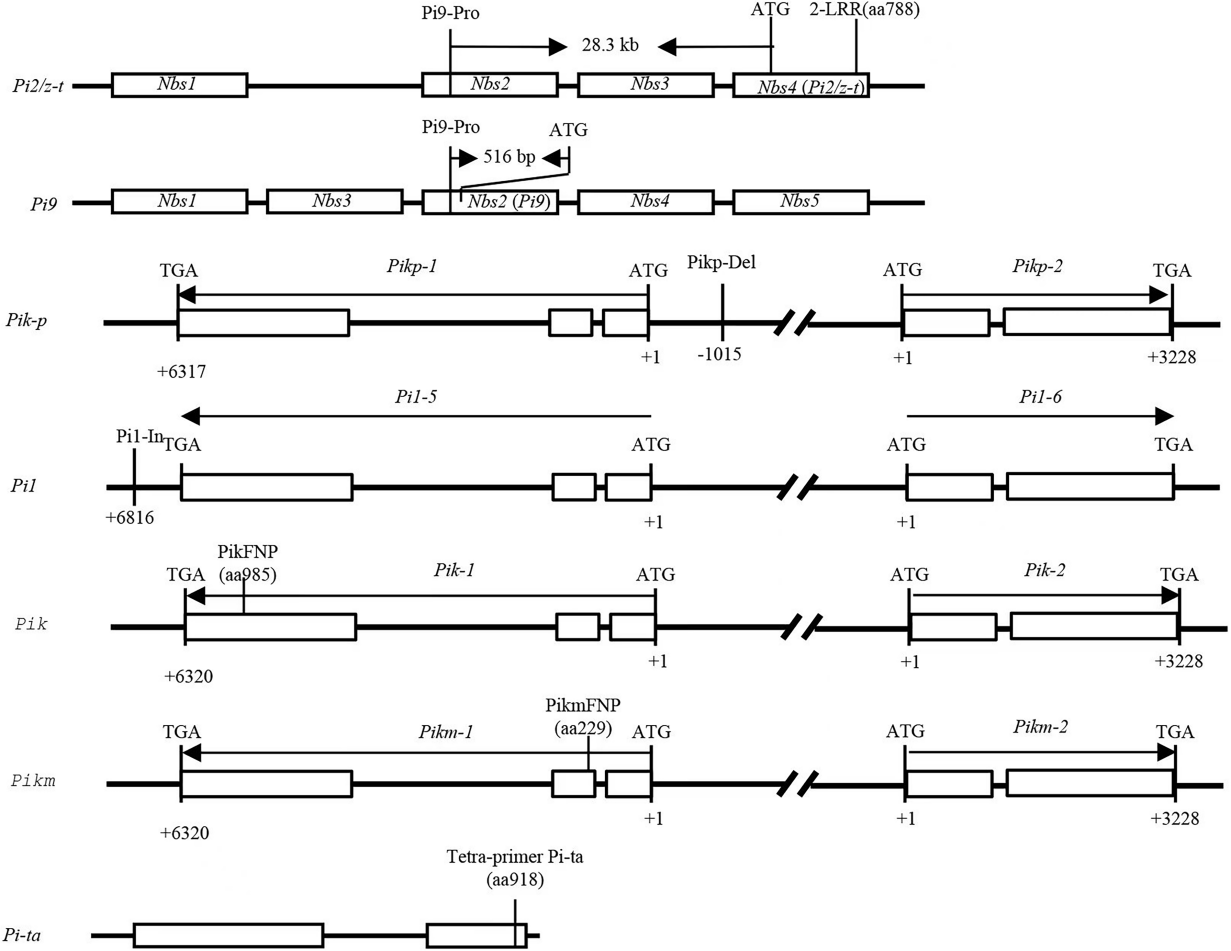
The physical maps of nine genes and their molecular markers in the study. The position of the nine molecular markers on the corresponding genes.

DNA from rice plant leaves were isolated using the cetyltrimethylammonium bromide (CTAB) method with some modifications (Tai and Tanksley, 1990). DNA from *M. oryzae* was extracted using the DNeasy Plant mini kit (QIAGEN, Germantown, MD). Six major *AVR* genes and nine *R* genes were validated with the markers listed in Supplementary Table 2. PCR amplification was carried out in a 25 μl reaction volume containing 20–200 ng template DNA, 1 μl of each forward and reverse primers (10 mM), 12.5 μl 2 × Mix buffer (Mg^2+^plus), 0.2 μl Taqase (5 U/ml), and 10.0 ul ddH_2_O. The PCR program was set up as follows: initial denaturation of 5 min at 94°C; followed by 32 cycles of denaturation for 30 s at 94°C, primers annealing for 30 s at varied temperatures according to the specific marker, and extension for 30 s at 72°C, followed by a final extension for 10 min at 72°C. For scoring the marker loci, the amplified PCR products of markers Pi9-Pro, Pikp-Del, Pi1-In, and Sepigm-4 were separated by 8% polyacrylamide gel electrophoresis along with a DL500 DNA ladder stained with silver staining. After electrophoresis, the gels were documented under UV using gel documentation system (AlphaImager, United States). PCR amplicons from the CAPS markers 2-LRR, PikFNP, and PikmFNP were digested with the restriction enzyme *PstI*, *Kpn*I, and *Mbo*I, respectively. The digested products were separated by electrophoresis with 8% polyacrylamide gels with subsequent visualization by silver staining. PCR products from *M. oryzae* were sequenced using an ABI3730 XL automatic DNA sequencer. DNA sequences were aligned using Clustal Omega (Sievers et al., 2011). Those PCR products with double-peak sequences were cloned into pMD18-T Vector (Takara, Japan) for further analysis. Figure 1 and Supplementary Table 2 list detailed information regarding the markers.

## Results

### Correlation between the field blast resistance and the bred periods of the 117 rice cultivars

A total of 117 rice cultivars were collected from the major rice-producing areas in China: 17 were from Guangdong, 23 from Fujian, 22 from Hunan, 6 from Jiangsu, 4 from Guangxi, 25 from Sichuan, 12 from Zhejiang, and 8 from other areas (Supplementary Table 1). These cultivars were evaluated for resistance to rice blast disease under field conditions in Shanghang, Fujian.

**Figure 2:**
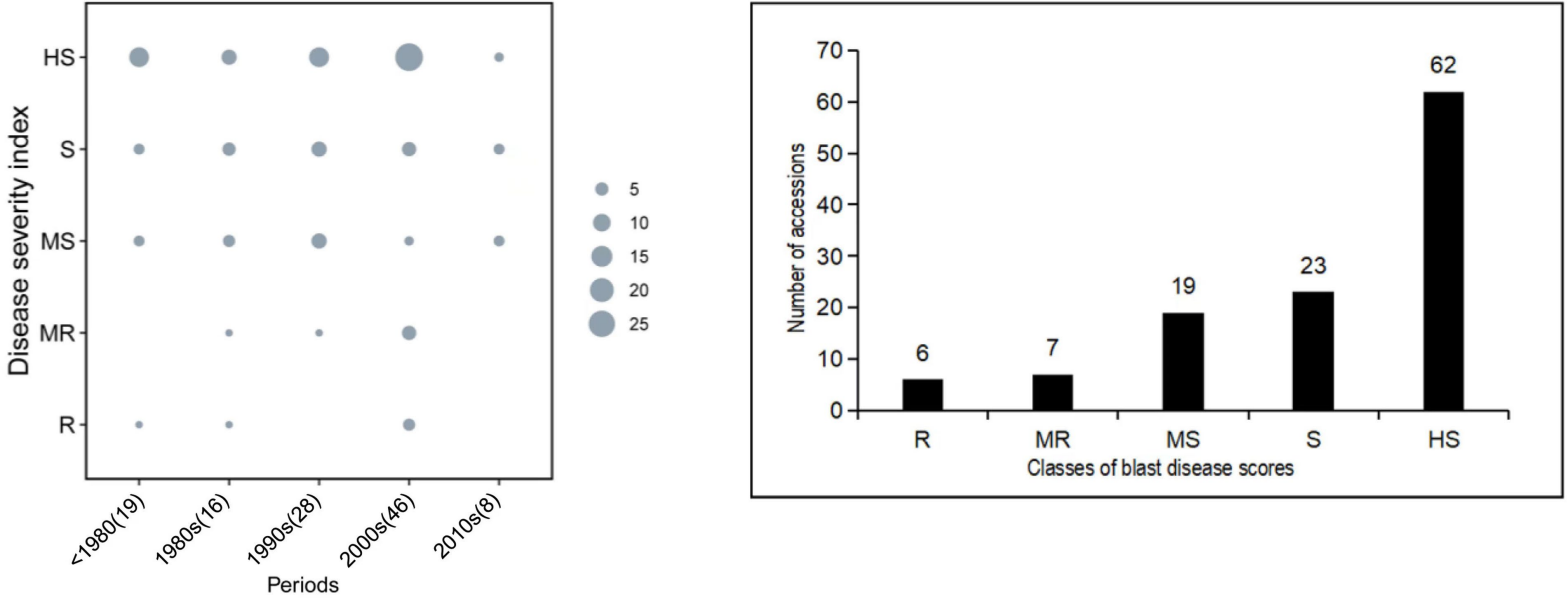
Distribution of blast-resistance scores for the 117 cultivars in the field test. Period: A, >1980; B, 1980s; C, 1990s, D, 2000s; E, 2010s. HR, highly resistant; R, resistant; MR, moderately resistant; MS, moderately susceptible; S, susceptible; HS, highly susceptible.

Figure 2 displays the distribution of blast disease severity scores from the field evaluation. The Pearson’s correlation values between the seedling stage and maximum tillering stage, seedling stage and fully ripe stage, and maximum tillering stage and fully ripe stage were 0.907, 0.596, and 0.604, respectively (*p*-value < 0.01; Supplementary Table 1), indicating a close relationship among the data acquired for the seedling stage and maximum tillering stage. The S, MS, and HS accessions were evenly distributed in every period of time, and the number of R, MR, MS, S, and HS cultivars changed from 6 to 62 with a rising tendency. Combining the results of blast disease severity and bred periods of time, there was no significant correlation between them (Figure 2). The percentage of MR, R, and HR cultivars changed from 5.26%, 12.5%, 3.57, 21.74%, and 0 across the past five decades with a random tendency (Supplementary Table 1), indicating that blast resistance among these rice cultivars has not obviously improved over time.

### The identification and distribution of nine rice blast *R* genes across the periods of time

**Figure 3:**
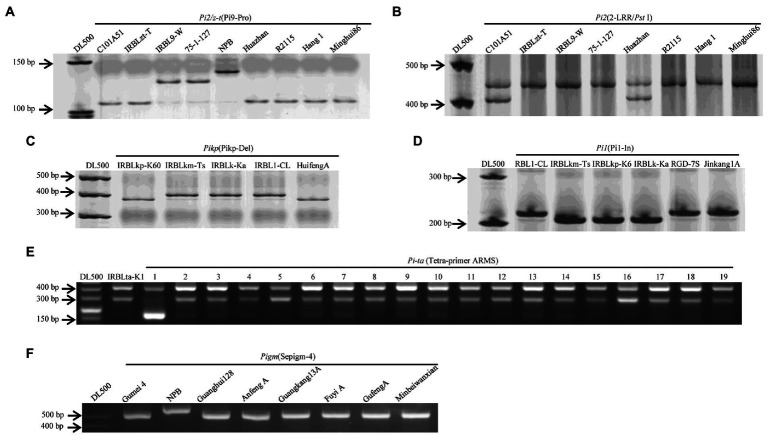
Electrophoretic gel of nine genes containing in the 117 cultivars. **E**, Number in *Pi-ta*: Positive control, IRBLta-K1; 1, NPB: Nipponbare; 2, Wanlixian; 3, Shuhui527; 4, Huazhan; 5, Minhui 3301; 6, Jiayu293; 7, RGD-7S; 8, Nanhui 511; 9, Shuhui 316; 10, Shen 08S; 11, Zhefu 802; 12, Duoxi1; 13,Ezao11; 14, Minghui82; 15, Shuhui 881; 16, Zhongxiang 1; 17, Chenghui 3203; 18, Lehui 188; 19, R2155. The other gene-containing cultivars in **A**–**D** and **F** are showen in gel.

Specific markers for *R* genes *Piz-t/Pi2*, *Pi9, Pi1, Pigm, Pik-p, Pik, Pik-m,* and *Pit-a* are shown in Figure 1. The frequency of the 9 major rice blast *R* genes ranged from 0 to 18 among the 117 cultivars (Figure 3). The presence of *Pi9* and *Piz-t/Pi2* was determined based on amplicons of 128 bp and 111 bp, respectively, using the allele-specific marker Pi9-Pro along with the positive control IRBL9-W/75–1-127 as well as IRBLzt-t/C101A51. *Pi9* was absent in all 117 cultivars. The PCR results for *Piz-t* and *Pi2* revealed amplicons of 439 and 399 bp, respectively, corresponding to the respective positive controls IRBLZT-t and C101A51. The results revealed the presence of *Pi2* in one variety and *Piz-t* in three cultivars (Figure 3). Additionally, five cultivars Minbeiwanxian, Guangkang 13A, Gufeng A, Fuyi A, and Guanghui 128 were identified to contain *Pigm* (Zeng et al., 2018). For the *Pik* locus, the presence of *Pik*, *Pikp*, and *Pikm* was determined using the dCAPS markers T1-2944G and T1-786A/G plus the markers A2-1879G and C1-685A/G corresponding to the positive control IRBLZK-Ka as well as IRBLZKp-k60 and IRBLKm-Ts, respectively (Zhai et al., 2011). Furthermore, two cultivars RGD-7S and Jinkang1A contained *Pi1,* and only one variety of Huifeng A had *Pikp* (Figure 3), and all other cultivars were negative for both *Pik* and *Pikm*. The presence of *Pi-ta* was determined based on amplicons of 286 and 406 bp (Zhang et al., 2013), which correspond to the positive control IRBLta-K1. *Pi-ta* was found in 18 cultivars with 286/406 bp (Figure 3). Taken together, the two cultivars Huazhan and R2115 possessed 2 of the positive alleles out of 6 *R* genes, and 30 cultivars had positive bands for a single *R* gene.

Through identifying the 117 cultivars based on the nine *R* genes specific primer sets, *Pi-ta, Pigm, Piz-t, Pik-p, Pi2,* and *Pi1* were revealed to present in 18, 6, 3, 1, 1, and 2 cultivars, respectively (Figure 3). *Pi-ta* and *Pigm* were distributed at a comparable level in these periods among the identified genes, further to find the cultivars in the 2000s had the most number of *R* genes among the five periods. However, the irregular application of *R* genes was present in modern cultivars at low frequency, suggesting that they just could be randomly utilized in breeding program.

### Haplotypes variance of six *AVR* genes in the *Magnaporthe oryzae* isolates corresponding to *R* genes

**Figure 4:**
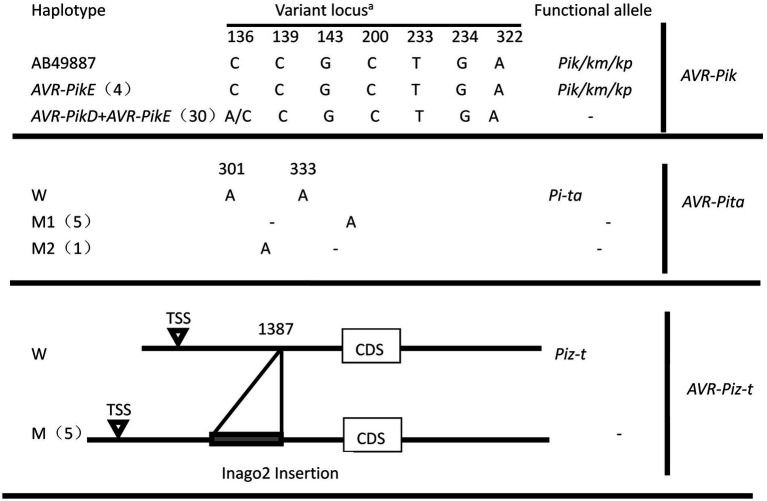
Characterization of allelic variations at AVR-Pik, AVR-Pita, and AVR-Piz-t. ^a^The positions were based on the reference sequence of *AVR-PikD* (AB498875)*, AVR-PikE* (AB498879), *AVR-Pita* (AF207841), *and AVR-Piz-t*(EU837058). **-**, avirulent genes without resistance genes; W, wide type; M, M1, and M2, mutant type.

The haplotypes of six *AVR* genes were determined using PCR and amplicon sequencing. PCR amplification utilized primer pairs (Supplementary Table 2) targeting the coding and promoter regions of the *AVR* genes, revealing three patterns: amplification yielded a product with the expected size, the unexpected size, or no amplification. *AVR-Pik* was amplified in 34 of these isolates, and the *AVR-Pita*, *AVR-Piz-t*, and *AVR-Pi9* fragments were present in 18, 10, and 17 isolates, respectively. Neither *AVR-Pia* nor *AVR1-Co39* was amplified in these isolates. Amplicon sequencing revealed that the *AVR-Pi9* sequence was identical to that of the avirulent strain KM004023, as described previously (Wu et al., 2015). The two haplotypes of *AVR-Pik*, namely *AVR-Pik-E* (AB498879) and its combination with *AVR-Pik-D* (AB498875), were identified in 4 and 30 isolates, respectively (Figure 4). Notably, *AVR-Pik-E* was identified in 4 isolates of the same diseased plant (Table 1). The *AVR-Pita* sequences in 18 isolates were compared with that of *AVR-Pita* in an avirulent strain (AF207841), revealing a loss of two nucleotides in the coding region (301A, and 333A) in two isolates (Figure 4; Table 1) that resulted in the loss of function of the avirulence effect of *AVR-Pita*. The inclusion of the promoter region in the PCR validation assay enabled the identification of a solo-LTR (Inagos homolog) insertion in the promoter of five *AVR-Piz-t* genes (Figure 4; Table 1).

**Table 1:** Analysis of different avirulence (*AVR*) genes in 35 rice blast isolates.

Diseased spike	Isolates	*AVR-Pi9*	*AVR-Piz-t*	*AVR-Pik*	*AVR-Pita*	*AVR-Pia*	*AVR-Co39*
1	SH1-1	-	-	D + E	W	-	-
	SH1-2	-	-	D + E	M1	-	-
	SH1-4	W	-	D + E	W	-	-
	SH1-5	W	-	D + E	M1	-	-
2	SH2-2	W	M	D + E	-	-	-
3	SH3-1	W	-	D + E	M1	-	-
	SH3-2	W	-	D + E	W	-	-
4	SH4-1	W	W	E	-	-	-
	SH4-2	W	W	E	-	-	-
	SH4-3	-	W	E	W	-	-
	SH4-4	W	W	E	-	-	-
	SH4-5	-	W	E	-	-	-
5	SH5	W	M	D + E	-	-	-
6	SH6	-	-	D + E	-	-	-
7	SH7-2	-	-	D + E	W	-	-
	SH7-3	W	-	D + E	W	-	-
	SH7-4	-	-	D + E	W	-	-
	SH7-5	W	-	D + E	W	-	-
8	SH8-1	-	-	D + E	W	-	-
	SH8-3	-	-	D + E	W	-	-
9	SH9-3	-	-	D + E	-	-	-
10	SH10-1	W	-	D + E	-	-	-
	SH10-2	W	-	D + E	-	-	-
	SH10-4	-	-	D + E	-	-	-
	SH10-5	W	-	D + E	-	-	-
	SH10-6	-	-	D + E	-	-	-
11	SH11-1	W	M	D + E	-	-	-
	SH11-3	-	M	-	-	-	-
	SH11-4	-	M	D + E	-	-	-
12	SH12-1	-	-	D + E	M1	-	-
	SH12-3	W	-	D + E	W	-	-
	SH12-4	-	-	D + E	M2	-	-
	SH12-5	W	-	D + E	M1	-	-
**13**	SH13-1	-	-	D + E	-	-	-
	SH13-5	-	-	D + E	W	-	-

W, wild type; M, mutant; D and E are two haplotypes of AVR-PikD and AVR-PikE, -, no PCR product. M1 and M2 indicate the loss of an A base in the coding region of AVR-Pita.

In total, 2, 1, and 1 novel variants were found in *AVR-Pita, AVR-Pik,* and *AVR-Piz-t* haplotypes, respectively (Figure 4), among which the 2 new haplotypes of *AVR-Pita* were distributed in 5 and 1 isolates, respectively. The new haplotypes of *AVR-Piz-t* were comprised 5 isolates of 3 diseased rice plants. Whereas the combination of *AVR-Pik-D + AVR-Pik-E* of *AVR-Pik* was comprised 85.7% isolates and widely distributed in 12 diseased rice plants (Table 1).

### Association analysis between the *AVR* genotypes in *Magnaporthe oryzae* and the field resistance of cultivars containing cognate *R* genes in rice

As the pathogenicity of the isolates depends on the haplotypes of both the *AVR* genes and corresponding *R* genes, field blast resistance of these 117 cultivars was identified in the disease nursery bed in Shanghang. All of these cultivars were scored for resistance in 2017. The correlation between the haplotypes of AVR genes and the field resistance of those cultivars containing cognate R genes was analyzed. Nearly all of the rice cultivars containing *Piz-t* or *Pi-ta* were susceptible to the field isolates in the test, it was possible that these variants of *AVR-Piz-t* and *AVR-Pita* led to loss of function of *Piz-t* and *Pi-ta*, respectively. Whereas 3 *Pik* cultivars that contain *Pi1* and *Pik-p* were HS to MR. In contrast, those cultivars possessing *Pi2* and *Pigm* exhibited good resistance to the *M. oryzae* isolates from the Fujian fields (Supplementary Table 2). Additionally, a number of cultivars such as Chenghui 178 and Neihui 99-14 that lacked a identified *R* gene expressed resistance, which suggests that other blast R genes in these cultivars may play important roles in resisting blast (Supplementary Table 1).

### Disease reaction of mono-genic lines against the local field blast isolates

The above data show that those cultivars containing *Pi2*, *Pi9*, and *Pigm* express broad-spectrum resistance in the field test, and few of functional genes in *Pik* locus were existed in these materials. Thus, 10 mono-genic lines containing these *R* genes and Co39 were inoculated with 14 local blast isolates, including isolates with *AVR-Piz-t*, *AVR-Pik-E, AVR-Pik-D + AVR-Pik-E*, and *AVR-Pi9,* etc. The isolate of SH4-4 containing *AVR-Piz-t* and *AVR-Pik-E* showed avirulence to *Piz-t* and *Pik/km/kh* in the pathotype test (Table 2), reiterating a perfect correlation between the existence of *AVR* and their cognate *R* genes. However, those mono-genic lines containing *Pi2, Pi9,* and *Pigm* were resistant to all of these isolates except that Gumei 4 was susceptible to SH10-6, suggesting that these three *R* genes express broad-spectrum/high resistance (Table 2). Additionally, as at least 1 isolate containing *AVR-Pik-D + AVR-Pik-E* exhibited MS, S, or HS to those mono-genic lines containing functional alleles of the *Pik* locus, suggesting that *AVR-Pik-D + AVR-Pik-E* was virulence to these genes (Table 2).

**Table 2:** Pathotypes of 10 mono-genic lines against 14 representative local isolates.

Isolates	Reactions of mono-genic lines to the isolates										
		*Piz* locus				*Pik* locus					
	Co39^a^	IRBLzt-t (*Piz-t*)	IRBL9-W/75–1-127 (*Pi9*)	C101A51 (*Pi2*)	Gumei4(*Pigm*)	IRBLZK-Ka (*Pik*)	IRBLKM-Ts (*Pikm*)	IRBLZKp-K60 (*Pikp*)	IRBL1-CL (*Pi1*)	IRBLkh-K3 (*Pikh*)	IRBLks-F5 (*Piks*)
SH1-4	S	S	R	R	R	R	-	-	R	R	-
SH1-5	S	S	R	R	R	R	R	R	R	R	R
SH3-1	S	S	R	R	R	R	R	R	MR	R	R
SH3-2	S	S	MR	R	R	R	S	R	R	R	S
SH4-4	MS	R	R	R	R	R	R	MS	R	R	R
SH6	S	S	R	R	R	R	R	R	R	R	R
SH7-3	S	S	R	R	R	R	R	R	R	R	S
SH8-1	S	S	R	R	R	R	MR	S	MR	MR	S
SH8-3	S	S	R	R	R	R	S	MR	R	S	S
SH9-3	S	S	R	R	R	-	S	MS	HS	-	-
SH10-5	S	S	R	R	R	-	S	S	MR	-	-
SH10-6	S	S	R	R	S	MS	MS	S	R	R	S
SH11-4	S	S	R	R	R	R	MS	S	MR	R	S
SH12-3	S	S	R	R	R	R	R	R	R	R	R

a Negative control.

HS, highly susceptible; S, susceptible; MS, moderately susceptible; R, resistant; MR, moderately resistant. The mono-genic lines were named in numeric order as follows: CO39 for the susceptible check. -, absent.

## Discussion

Rice is one of the most important food crops in Fujian, accounting for 78% of the planting area and 80% of the total grain output (Huang et al., 2012). The diverse geography and variable climate of the Fujian region have stimulated the adaptive variation of *M. oryzae* in rice fields, and *R* genes in rice varieties have influenced the formation of the variation of *M. oryzae* genotypes (Selisana et al., 2017). Thus, monitoring the *R* genes in rice cultivars and *AVR* genotypes of *M. oryzae* is critical for effective utilization of *R* genes to improve rice yields. However, little effort has been directed toward the simultaneous characterization of both *R* genes of rice and A*VR* genes of *M. oryzae* (Kolmer and Hughes, 2018).

Conventional pathogenicity analyses of *M. oryzae* populations and the evaluation of resistance levels of rice varieties have been carried out in blast nurseries or by pathotyping in a greenhouse, and differential varieties have been derived from cultivars LTH and Co39-IRTL (Telebanco-Yanoria et al., 2008), but such analyses are both time-consuming and laborious (Vasudevan et al., 2014). Recently, PCR-based gene diagnosis has been applied to infer the effectiveness of *R* genes, and this approach has been shown to be suitable for large-scale pathogen identification because the PCR method does not require live *M. oryzae* strains (Imam et al., 2015). The present study categorized 117 cultivars into five group at 10-year level of planting period. Our results showed that the genotype distributions for nine *R* genes were randomly distributed in these periods based on their corresponding markers (Figures 1, 3). As a matter of fact, most of the rice cultivars in the early decades possessed few R genes, and although more recent cultivars such as in 2000s possess more *R* genes, all express similar resistance to the field isolates as shown in Figure 2. The smaller class of blast disease scores HR consists of six cultivars, whereas the major group HS included 62 ones (Figure 2-right). Accordingly, avirulence genes of blast fungal were differentiated into wild and mutant genotypes by using sequencing analysis (Table 1). The corresponding disease reaction in mono-genic lines was found concurrent with the avirulent and virulent genes, respectively (Table 2). The consistent results in the study showed by the individual markers for selected blast-resistance genes and AVR genes make them a suitable marker for genotyping of rice blast resistant genes in the rice germplasm and avirulence genes in the blast fungal, respectively.

Some previous studies have revealed that the rice blast pathogen population, which are related to the divergence of *AVR* genes that have been shaped by changing environmental conditions, can have a clear impact on *R* gene-mediated resistance to blast disease (Li et al., 2019). Our data demonstrate that several mutational events were brought about at the *AVR-Pita* and *AVR-Piz-t* loci, leading to those cultivars containing *Pi-ta* and *Piz-t* exhibited susceptible to the field isolates. Additionally, previous studies revealed that *AVR-Pita* alleles are highly diversified, and transposon insertions, point mutations, frame-shift mutations, partial or complete deletions, and sequence variations in the coding or promoter region caused a change from avirulence to virulence of *AVR* genes (Xing et al., 2013; Chen et al., 2014). Similarly, the loss of function of *AVR-Piz-t* was also observed in 312 isolates collected from the rice-producing areas of Yunnan, and a 198-bp insertion homologous to solo-LTR of the retrotransposon inago2 in the promoter region of *AVR-Piz-t* in one isolate was found to have evolved from avirulence to virulence (Wang et al., 2020). Meantime, the changes of virulence frequencies of *M. oryzae* for *Pi-ta* and *Piz-t* happened in different years, suggesting that the disease reaction of rice cultivars were changing overtime (Shen, 2018).

Even though *Pikm*, *Pikh, Pikp, Pik,* and *Pi1* exhibited a high level of resistance to blast fungus from Fujian, Guangdong, Sichuan provinces and Japanese, etc. (Yang et al., 2008; Wang et al., 2017; Zhang et al., 2017; Tian et al., 2021), the stepwise evolution with the *AVR-Pik* allele found in the United States, Philippines, and Japanese isolates populations and attempt to overcome them (Raffaele et al., 2010; Kanzaki et al., 2012; Selisana et al., 2017; Wang et al., 2017). To date, six AVR-Pik variants (A–F) have been identified, and they differ in just five amino acid positions. The generation and diversity of mutational events may be influenced by introducing an *R* gene in new rice varieties (Zhou et al., 2007; Daverdin et al., 2012). *M. oryzae* isolates carrying AVR-PikD trigger immune responses in rice lines containing *Pikp/km/kh/Pik,* isolates carrying AVR-PikE only elicit a response in rice lines with *Pikm/kh*. *AVR-Pik* has evolved *via* gene duplication and substitution mutations in the coding regions of *AVR-Pik/km/kp* in *M. oryzae* populations (Wu et al., 2014; Li et al., 2019; Longya et al., 2019). However, no known *Pik* alleles respond to the combination effectors of AVR-PikD + AVR-PikE identified in the present study. Subsequently, the corresponding rice *R* genes may have become blind to the new genotypes (Kanzaki et al., 2012). Consistent with these results, most of those mono-genic lines carrying *Pikm/kp/ks/1* were partly susceptible to those isolates containing AVR-PikD + AVR-PikE; however, whether the combination of AVR-PikD + AVR-PikE would affect gene function needs to be further validated by transcript or translation level.

At least six functional genes, including *Pigm, Pi2*, *Pi9,* and *Piz-t*, reside within the *Pi2/9* locus. In contrast to the fact that *Piz-t* exhibited relatively lower resistance to *M. oryzae* in Fujian, China, *Pigm*, *Pi9,* and *Pi2* have been shown to confer higher and broad-spectrum resistance against a wide range of blast isolates present in this planting region (Jiang et al., 2012; Tian et al., 2016, 2020). Previous studies showed that *Piz-t* was the first widely used in the 1970s (Fjellstrom et al., 2006), but with the evolution of AVR-Piz-t, the gene has lost its resistance (Marchetti et al., 1987; Conaway-Bormans et al., 2003; Tian et al., 2016); whereas *Pi2* and *Pigm* were distributed in recent rice varieties from Fujian and Guangdong as they exhibited good resistance to rice blast from the two provinces (Tian et al., 2020); Additionally, Vasudevan et al. (2014) identified 289 accessions containing *Pi2* from a total collection of over 120,000 ones originating from 13 major rice-growing countries showed broad-spectrum resistance against all five single rice blast isolates. In contrast, *Pi9* is not widely utilized in Chinese rice varieties. In our study, the rice cultivars with *Pi2, Pi9,* and *Pigm* can be classified *R* or *MR*, whereas those cultivars containing *Piz-t* were relatively more susceptible to disease. Further to find that those mono-genic lines carrying *Pi9*, *Pi2,* or *Pigm* exhibited good resistance to the local isolates. Intriguingly, the association between the AVR gene(s) and the disease reaction was not completely understood in our study, such as some isolates containing AVR-Pi9 show negative band by gene amplification, which could be explained by addition of more number of markers needs to be tested for new *R* genes or Avr genes. All these aforementioned findings provide valuable insights pertaining to the selection of desirable *R* genes-particularly those with durable resistance.

## Conclusion

In the study of nine *R* genes in 117 representative cultivars over past 5 decade years and six cognate *AVR* genes in 35 *M. oryzae* isolates. Nine *R* genes have not been conscious utilization in these periods of time. The three observed variants of *AVR-Pita, AVR-Piz-t,* and *AVR-PikD + AVR-PikE* confer virulence to their cognate *R* genes, which may be explained by the rapid evolution of local *M. oryzae* isolate in respond to these genes. Our results also indicated that effective control of blast disease will require the functional allele of *Pi2/9* locus.

## Data availability statement

The raw data supporting the conclusions of this article will be made available by the authors, without undue reservation.

## Author contributions

DT conceived the initial screening and research plans. DT, LY, and HZ designed the experiments. YD, XY, GL, QL, ZC, XG, and YS performed most of the experiments and analyzed the data. DT wrote the manuscript with the contributions of all the authors. All authors contributed to the article and approved the submitted version.

## Funding

The work was funded by the Key Program of the National Natural Science of Fujian province (2022J02010), the Youth Program of Fujian Academy of Agricultural Sciences (YC2019004), the ‘5511’ Collaborative Innovation project for High-quality Development and Surpasses of Agriculture between Government of Fujian Province and Chinese Academy of Agricultural Sciences (Grant no. XTCXGC2021002), the Opening Foundation of Jiangsu Key Laboratory for Eco-Agricultural Biotechnology around Hongze Lake (HZHLAB2101), and the Natural Science Foundation of Jiangsu Provincial Department of Education (17KJA180002 and 19KJB180011).

## Conflict of interest

The authors declare that the research was conducted in the absence of any commercial or financial relationships that could be construed as a potential conflict of interest.

The reviewer YD declared a shared affiliation with several of the authors, DT, XY, ZC, and XG, to the handling editor.

## Publisher’s note

All claims expressed in this article are solely those of the authors and do not necessarily represent those of their affiliated organizations, or those of the publisher, the editors and the reviewers. Any product that may be evaluated in this article, or claim that may be made by its manufacturer, is not guaranteed or endorsed by the publisher.

## Supplementary material

The Supplementary material for this article can be found online at: https://www.frontiersin.org/articles/10.3389/fmicb.2022.1007492/full#supplementary-material

Supplementary Table 1 Primers used for PCR diagnosis of avirulence and resistance genes.

Supplementary Table 2 List of 117 cultivars bred over five decades periods of time that were used for field evaluation of *M. oryzae*.
