# Supplementary material for: Association analysis of rice resistance genes and blast fungal avirulence genes for effective breeding resistance cultivars
Source: Front Microbiol. 2022 Nov 9;13:1007492. doi: 10.3389/fmicb.2022.1007492 (PMC9682276; doi:10.3389/fmicb.2022.1007492)
Supplement: Supplementary Table 1 — Primers used for PCR diagnosis of avirulence and resistance genes. [file Table_1.DOCX]

| **Cultivars** | **Periods^a^** | **Provinces** | **Disease scoring at the three stages** | | | **Comprehensive resistance^b^** |
| --- | --- | --- | --- | --- | --- | --- |
|  |  |  | **Seedling** | **Tillering** | **Mature** |  |
| Aijiaonante | A | Guangdong | 9 | 7 | 9 | HS |
| Aituogu151 | A | Sichuan | 7 | 5 | 9 | HS |
| Aizizhan | A | Guangxi | 7 | 5 | 9 | HS |
| Dongtingwanxian | A | Hubei | 7 | 5 | 5 | MS |
| Erjiunan1 | A | Zhejiang | 9 | 7 | 9 | HS |
| Ganzaoxian2 | A | Jiangxi | 7 | 7 | 7 | S |
| Guangchang13 | A | Guangdong | 7 | 5 | 9 | HS |
| Guangluai4 | A | Guangdong | 5 | 5 | 7 | S |
| Gui630 | A | Hunan | 7 | 5 | 9 | HS |
| Guichao2 | A | Guangdong | 5 | 5 | 7 | S |
| Jinnante43 | A | Guangxi | 7 | 5 | 9 | HS |
| Minbeiwanxian | A | Fujian | 0 | 0 | 1 | R |
| Nanjing11 | A | Jiangsu | 7 | 5 | 9 | HS |
| Nantehao | A | Jiangxi | 5 | 3 | 9 | HS |
| Taizhongzailai1 | A | Taiwan | 7 | 5 | 5 | MS |
| V20A | A | Hunan | 5 | 5 | 9 | HS |
| Wanlixian | A | Hunan | 7 | 5 | 9 | HS |
| Zhenshan97A | A | Sichuan | 5 | 5 | 9 | HS |
| Zhongnong4 | A | Sichuan | 7 | 5 | 5 | MS |
| BobaiA | B | Fujian | 9 | 7 | 7 | S |
| Ce64 | B | Hunan | 9 | 7 | 7 | S |
| FuyiA | B | Fujian | 3 | 3 | 1 | R |
| Guanghui128 | B | Guangdong | 0 | 0 | 3 | MR |
| Gui99 | B | Guangxi | 7 | 5 | 9 | HS |
| II-32A | B | Hunan | 5 | 5 | 9 | HS |
| Jin23A | B | Hunan | 9 | 7 | 9 | HS |
| Minghui63 | B | Fujian | 7 | 5 | 7 | S |
| Teqing2 | B | Guangdong | 7 | 5 | 9 | HS |
| Xiangwanxian1 | B | Hunan | 7 | 5 | 5 | MS |
| Xiangzaoxian7 | B | Hunan | 9 | 7 | 9 | HS |
| Yangdao2 | B | Jiangsu | 7 | 5 | 7 | S |
| Yinzhan | B | Hubei | 7 | 5 | 7 | S |
| Zhe852 | B | Zhejiang | 7 | 7 | 9 | HS |
| Zhefu802 | B | Zhejiang | 5 | 5 | 5 | MS |
| Zhongyu1 | B | Zhejiang | 7 | 5 | 9 | HS |
| 9311 | C | Jiangsu | 5 | 5 | 7 | S |
| Chenghui448 | C | Sichuan | 3 | 3 | 3 | MR |
| D702A | C | Sichuan | 9 | 7 | 9 | HS |
| Duoxi1 | C | Sichuan | 7 | 7 | 5 | MS |
| Ezao11 | C | Hubei | 7 | 5 | 9 | HS |
| Fuhui838 | C | Guangxi | 7 | 7 | 9 | HS |
| Guanghui998 | C | Guangdong | 5 | 5 | 7 | S |
| Guangzhan63S | C | Anhui | 7 | 7 | 9 | HS |
| Jiayu293 | C | Zhejiang | 5 | 5 | 9 | HS |
| LongtepuA | C | Fujian | 7 | 5 | 7 | S |
| Luhui17 | C | Sichuan | 7 | 5 | 7 | S |
| Minghui72 | C | Fujian | 5 | 5 | 5 | MS |
| Minghui77 | C | Fujian | 7 | 5 | 5 | MS |
| Minghui82 | C | Fujian | 7 | 5 | 9 | HS |
| Minghui86 | C | Fujian | 9 | 7 | 9 | HS |
| Peiai64S | C | Hunan | 7 | 5 | 5 | MS |
| R402 | C | Hunan | 7 | 5 | 7 | S |
| SE21S | C | Fujian | 9 | 7 | 9 | HS |
| Shuhui881 | C | Sichuan | 7 | 5 | 9 | HS |
| Wan3 | C | Hunan | 5 | 5 | 7 | S |
| Yangdao4 | C | Jiangsu | 5 | 5 | 7 | S |
| You1A | C | Hunan | 5 | 5 | 9 | HS |
| Yue4A | C | Hunan | 7 | 5 | 9 | HS |
| Zhe733 | C | Zhejiang | 9 | 7 | 9 | HS |
| Zhenxian 232 | C | Jiangsu | 7 | 5 | 5 | MS |
| Zhonghui8015 | C | Zhejiang | 7 | 7 | 5 | MS |
| Zhongjian100 | C | Zhejiang | 5 | 5 | 9 | HS |
| Zhongxiang1 | C | Zhejiang | 7 | 7 | 9 | HS |
| Chenghui 727 | D | Sichuan | 3 | 3 | 1 | R |
| Chenghui178 | D | Sichuan | 0 | 0 | 1 | R |
| Chenghui3203 | D | Sichuan | 5 | 3 | 1 | R |
| Chuanxiang29A | D | Sichuan | 9 | 7 | 9 | HS |
| D62A | D | Sichuan | 7 | 5 | 9 | HS |
| FengyuanA | D | Hunan | 5 | 5 | 7 | S |
| FueS1 | D | Fujian | 7 | 5 | 9 | HS |
| Fuhui718 | D | Sichuan | 7 | 5 | 5 | MS |
| Gang46A | D | Sichuan | 5 | 5 | 3 | MR |
| GD-5S | D | Guangdong | 5 | 5 | 9 | HS |
| Guangkang13A | D | Fujian | 0 | 0 | 3 | MR |
| GufengA | D | Fujian | 3 | 3 | 3 | MR |
| Hang1 | D | Fujian | 7 | 7 | 9 | HS |
| Huanghuazhan | D | Guangdong | 7 | 7 | 9 | HS |
| HuaxiangA | D | Sichuan | 9 | 7 | 9 | HS |
| Huazhan | D | Guangdong | 3 | 3 | 3 | MR |
| Jiayu164 | D | zhejiang | 5 | 5 | 9 | HS |
| Jiayu948 | D | Zhejiang | 7 | 5 | 9 | HS |
| Jinkang1A | D | Fujian | 0 | 3 | 9 | HS |
| Kangwenqingzhan | D | Guangdong | 5 | 5 | 9 | HS |
| Lehui188 | D | Sichuan | 7 | 5 | 9 | HS |
| Luxiang618A | D | Sichuan | 5 | 5 | 9 | HS |
| Mianhui725 | D | Sichuan | 9 | 7 | 7 | S |
| Minghui2155 | D | Fujian | 7 | 5 | 9 | HS |
| Minhui3119 | D | Fujian | 7 | 5 | 9 | HS |
| Minhui3139 | D | Fujian | 7 | 5 | 7 | S |
| Minhui3229 | D | Fujian | 9 | 9 | 9 | HS |
| Minhui3301 | D | Fujian | 9 | 5 | 9 | HS |
| Nanhui511 | D | Sichuan | 7 | 5 | 9 | HS |
| Neihui99-14 | D | Sichuan | 0 | 3 | 1 | R |
| R2115 | D | Hunan | 0 | 0 | 1 | R |
| RGD-7S | D | Guangdong | 0 | 0 | 3 | MR |
| Shen95A | D | Guangdong | 5 | 5 | 9 | HS |
| Shuhui498 | D | Sichuan | 5 | 5 | 9 | HS |
| Shuhui527 | D | Sichuan | 9 | 7 | 5 | MS |
| TaifengA | D | Guangdong | 7 | 7 | 9 | HS |
| TianfengA | D | Guangdong | 7 | 5 | 9 | HS |
| WufengA | D | Guangdong | 7 | 5 | 9 | HS |
| Xiangzaoxian45 | D | Hunan | 5 | 5 | 7 | S |
| Y58s | D | Hunan | 7 | 5 | 7 | S |
| Yanhui559 | D | Jiangsu | 7 | 7 | 9 | HS |
| Yixiang1A | D | Sichuan | 9 | 7 | 9 | HS |
| YuefengA | D | Guangdong | 5 | 5 | 5 | MS |
| Zhongzao23 | D | Zhejiang | 9 | 7 | 9 | HS |
| Zhou903 | D | Zhejiang | 9 | 7 | 9 | HS |
| ZhunS | D | Hunan | 7 | 5 | 9 | HS |
| 710S | E | Fujian | 7 | 7 | 7 | S |
| AnfengA | E | Fujian | 0 | 3 | 5 | MS |
| HuifengA | E | Fujian | 7 | 5 | 5 | MS |
| Longke638S | E | Hunan | 5 | 5 | 5 | MS |
| R1128 | E | Hunan | 9 | 7 | 7 | S |
| R900 | E | Hunan | 7 | 7 | 9 | HS |
| Shen08S | E | Guangdong | 7 | 5 | 7 | S |
| Shuhui316 | E | Sichuan | 3 | 3 | 9 | HS |

^a^ the bred periods A, >1980; B, 1980s; C, 1990s, D, 2000s; E, 2010s. ^b^HR, highly resistant; R, resistant; MR, moderately resistant; MS,moderately susceptible; S, susceptible; HS, highly susceptible
