# Supplementary material for: Association analysis of rice resistance genes and blast fungal avirulence genes for effective breeding resistance cultivars
Source: Front Microbiol. 2022 Nov 9;13:1007492. doi: 10.3389/fmicb.2022.1007492 (PMC9682276; doi:10.3389/fmicb.2022.1007492)
Supplement: Supplementary Table 2 — List of 117 cultivars bred over five decades periods of time that were used for field evaluation of M. oryzae. [file Table_2.DOCX]

| Genes(markers) | | **Primer sequence (5′- 3′)** | | Expected  size | Notes |
| --- | --- | --- | --- | --- | --- |
|  |  | **F primer** | **R primer** |  |  |
| *AVR* | *AVR-Pi9* (MW288376.1) | CCCATGTGTGCTTATCGCGTG | GACTTGAGAGAACTGCATGTC | 1787 | Promoter+CDS |
|  | *AVR-Piz-t* (EU837058) | GTTGCGATTATGATCCGTCG | GTACTCTAGCAAACGACCGG | 1144 | Promoter+CDS |
|  | *AVR-Pita* (AF207841) | CAGGCATACATTGGAGAGCC | CCCTCCATTCCAACACTAAC | 1549 | Promoter+CDS |
|  | *AVR-Pik* (AB498875) | TCCTGCTGCTAACTCCATTC | TCAACCAAGCGTAAACCTCG | 1200 | Promoter+CDS |
|  | *AVR-Co39*(AF463528) | TGCCGCATTTTGCTAACCG | GCGAATCCATAGACAAGGAC | 994 | Promoter+CDS |
|  | *AVR-Pia* (AB498873.1) | CAGAGAAACGGACTTGGAGG | GGTATACACGTACGGTAGGG | 1220 | Promoter+CDS |
| *R* | *Pi9* (Pi9-Pro) | TGATTATGTTTTTTATGTGGGG | ATTAGTGAGATCCATTGTTCC | 128 | Tian et al., 2016 |
|  | *Piz-t/Pi2*(2-LRR/Pst I) | CGTTGTATAGGACAGTTTCATT | AATCTAGGCACTCAAGTGTTC | 439/*Pst*I | Hua et al., 2015 |
|  | *Pikp* (Pikp-Del) | TGGTTAAATAGGACTCCCTC | CATTCGCAGACTCGTTGA |  | Tian et al., 2021 |
|  | *Pita* (Tetra-primer ARMS) | CCGTGGCTTCTATCTTTACTTG | CAAGTCAGGTTGAAGATGCATTGA | 286/406 | Zhang et al., 2013 |
|  |  | TGGTGCTGAAGGGAGAGACT | TTAGGGCCAACATTCTACGG |  |  |
|  | *Pigm* (Sepigm-4) | TCGCAGCCATCCAAAGTGAGTC | TACACCTGCGAATCAAATCACT | 513 | Zeng et al., 2018 |
|  | *Pi1* (Pi1-In) | TGAGGTAGAAGCGGGAGC | GGTTGGTCGAAACCAGAA |  | Tian et al., 2021 |
|  | *Pik* (PikFNP) | TTCGAGGCCCTACCAAGACA | CATGGAAGGCTATCCTTGGTA | 103/*Kpn*I | Zhai et al., 2011 |
|  | *Pik-m* (PikmFNP) | TCGCCGGTGACCTAAGAGAT | GATTTCACCGGCGCAAGCAT | 150/*Mbo*I | Zhai et al., 2011 |
